# Supplementary figures and images for: Looking under the hood of a hybrid two-way texting intervention to improve early retention on antiretroviral therapy in Malawi: an implementation fidelity evaluation
Source: Implement Sci. 2025 Jan 22;20:6. doi: 10.1186/s13012-025-01418-7 (PMC11753095; doi:10.1186/s13012-025-01418-7)

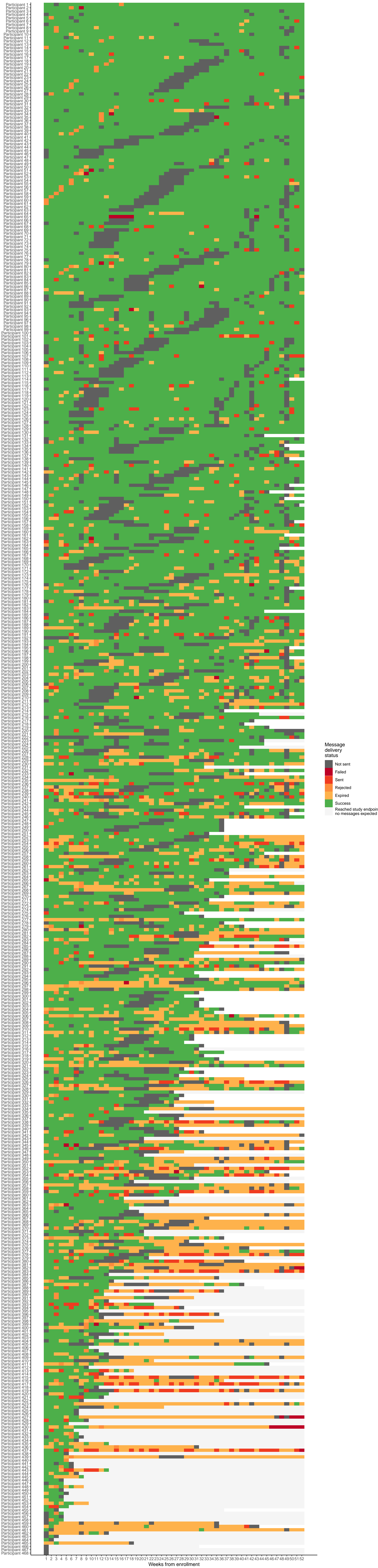

Supplement: Supplementary file 2 — Supplementary Material 2: Appendix 2. Heat map of motivation message delivery per participant per week throughout follow-up. [file 13012_2025_1418_MOESM2_ESM.pdf]
